# Evaluation of Salivary Level of Heat Shock Protein 70 in Patients with Chronic Periodontitis

**DOI:** 10.30476/DENTJODS.2020.87080.1228

**Published:** 2021-09

**Authors:** Paria Motahari, Solmaz Pourzare Mehrbani, Hamed Jabbarvand

**Affiliations:** 1 Dept. of Oral Medicine, Faculty of Dentistry, Tabriz University of Medical Sciences, Tabriz, Iran

**Keywords:** Chronic periodontitis, HSP70 Heat-Shock Proteins, Saliva

## Abstract

**Statement of the Problem:**

Traditional clinical criteria are usually not sufficient for determining the sites of active periodontal disease, monitoring the response to treatment, or measuring the susceptibility to future disease development. Past studies have shown that heat shock protein 70 (HSP70) are involved in the etiology of periodontal disease.

**Purpose:**

The aim of this study was to evaluate the level of HSP70 in saliva of patients with chronic periodontitis (CP).

**Materials and Method:**

In our case-control study, the saliva samples of 45 patients with CP and 45 age- and sex-matched healthy subjects were collected. Salivary HSP70 was measured by enzyme-linked immunosorbent assay method. The results were analyzed by statistical tests using SPSS 16 and the statistically significant difference was set at *p*< 0.05.

**Results:**

In this study, the mean salivary HSP70 level was 2.81±0.61ng/ml in the patient group and 1.96±0.77ng/ml in the healthy group, with a significant difference (*p*< 0.05). In addition, the results of spearman correlation analysis showed a positive correlation between salivary HSP 70 and clinical periodontal index.

**Conclusion:**

The results of this study showed that the salivary HSP70 level in patients with CP is higher than that in healthy subjects. As a result, salivary HSP70 might be considered as a marker in the pathogenesis of CP.

## Introduction

Periodontitis is a disease that affects dental support structures. Periodontitis can be divided in three main forms: chronic, aggressive and as a manifestation of a systemic disease. Chronic periodontitis (CP) as the common form of this disease, generalized form involves more than 30% of dentition [ 1 - 2 ]. Periodontitis is an inflammatory disease in which the infiltration of mononuclear cells into the gingival tissue results in connective tissue and alveolar bone resorption. Although periodontal bacteria are agents of periodontitis, progression and severity of periodontal disease are determined by the host immune response [ 3 - 4 ]. The exact mechanism of periodontal tissue destruction has not yet been clarified. Immunohistochemical studies have shown that the expression of heat shock proteins (HSPs) in the basal layer of periodontal pockets is positive. Moreover, there is an increase in infiltration of mononuclear inflammatory cells below the basal layer of periodontal pockets. Thus, periodontal bacteria stimulate the periodontal cells to increase expression of HSPs, thereby stimulating macrophages and other inflammatory cells to produce pro-inflammatory cytokines, a mechanism that is involved in tissue destruction of periodontal disease [ 1 - 5 ].

HSPs are called proteins that are expressed in stressful conditions in the cell. The role of these proteins is to prevent the transformation of proteins under stress. These proteins are present in all living cells in the attached or unbound state of the proteins. HSPs are involved in the function of immune cells. These proteins are divided into five major classes, one of which is HSP70 [ 6 - 7 ]. HSP70 has intracellular and extracellular activities including cytoprotection and immune modulation response. Due to its protective role and inhibition of apoptosis, HSP70 protects cells from tissue destruction [ 8 - 11 ]. 

According to previous studies, HSPs levels, especially HSP70, are elevated in patients with CP [ 12 - 14 ]. Traditional clinical criteria are usually not sufficient to determine the sites of active periodontal disease, monitoring the response to treatment, or to measure the susceptibility to future disease development [ 1 - 3 ]. Saliva is an important source of clinical information and is considered a mirror of oral health because it has specific markers for the periodontal disease [ 15 ].

Given that all of studies have been performed on serum and tissue of patients with CP and since saliva preparation is non-invasive method, the main purpose of this paper was to assess the level of HSP70 in saliva of patients with CP.

## Materials and Method

In this case-control study, we compared the salivary level of HSP70 in patients with CP and healthy controls.

In the case group, patients with moderate or severe generalized CP (N= 45) were selected from the all patient who referred to the Department of Oral Medicine and Periodontology, Tabriz Faculty of Dentistry from October 2018 to June 2019. 

Inclusion criteria were general health, CP diagnosis according to the International Workshop for a Classification of Periodontal Diseases [ 16 ], and agreement with examination. The exclusion criteria integrated history of diabetes, cardiovascular disorders, immunodeficiency, cancer, smoking and current lactation, or pregnancy. 

The control group (N = 45) were randomly selected in the same time and matched with the CP group in terms of gender and age. All controls had at least 20 teeth without any history or clinical signs of periodontitis and gingivitis. They were generally healthy people who agreed with examinations. Exclusion criteria were the same as those used with patients with CP. 

The assessed parameters were bleeding on probing (BOP), clinical attachment loss (CAL), probing pocket depth (PPD) and radiographs. PPD and CAL were assessed using a William's probe from six sites on each remaining teeth. At least 30% of sites must have PPD>5mm and CAL>3mm. BOP was recorded using the bleeding point index [ 17 ] at 6 points for each teeth and a bleeding percentage was calculated for each patient. All patients should have no history of periodontal therapy or antibiotic treatment for at least 3 months before participating in the study.

### Saliva sampling

Saliva sampling was performed using NAVAZESH method [ 18 ]. Regarding this method, participants did not eat or drink anything two hours before sampling. Fifteen minutes before sampling, the volunteers washed their mouths, and then their oral cavity was examined with mirror under adequate light to ensure that no material was remained in their oral cavity. The patient's saliva samples were collected within 16-20 minutes using sterile disposable plastic container and transferred to the laboratory immediately. The laboratory samples were then centrifuged, the granular particles were discarded, and the supernatant was partitioned into a micro tube and then kept at -70°C for analysis.

### Enzyme linked immunoassay for HSP70

Salivary HSP70 level was evaluated by commercial enzyme-linked immunosorbent assay kit (HSP70 ELISA Kit-ESK-715, Assay Designs Inc, Ann Arbor, Michigan). Prior to measurement, the samples of saliva were defrosted and centrifuged at 10,000 rpm for 1 minute.

### Statistical analysis of data

The level of salivary HSP70 in both groups was measured in this study by enzyme-linked immunosorbent assay method. The parameters of analysis were reported using descriptive statistics (Mean±Standard deviation). In addition, comparisons between the studied groups were performed using an independent t-test for variables with a normal distribution and a Mann-Whitney test for those with a non-normal distribution. The medians of salivary HSP70 in the non-periodontitis group were used for setting the threshold as well. To examine the diagnostic potential of salivary HSP70, logistic regression model was used to determine odds ratio and 95% confidence intervals. The correlation between clinical parameters and salivary HSP70were evaluated using the Spearman’s rank correlation coefficient. The software used in this study was SPSS 16 and statistically significant difference was considered when *p*< 0.05.

### Ethical considerations

The Research Ethics Committee of Tabriz University of Medical Sciences has approved this study (IR. TBZME-D.REC.1397.913). The participants in this study were consented and only necessary interventions were performed for them. Therefore, this study had no adverse effects on patients and their therapeutic process. 

## Results

Table 1 lists the means and standard deviations of salivary levels of HSP70 in CP and control groups. This Table also shows the demographic and clinical characteristics of the groups as well as *p* Values. According to our analysis, there was a significant difference in salivary HSP70 level between these groups (*p*<0.05). As shown in Table 2, based on logistic regression analysis, the probability of high salivary HSP70 level in patients with CP is 10 times higher than that in healthy individuals, which is statistically significant indicating the high diagnostic potential of this biomarker in CP.

**Table 1:** Demographics and clinical parameters and salivary HSP70 levels in the case and control groups

Parameters		Case(N=45)	Controls(N=45)	*p* Value
Age (Mean± Standard deviations)		37.5±7.3	35.8±8.3	0.29
Gender	Female	21	21	-
	Male	24	24	-
PPD (Probing Pocket Depth) (mm)		3.05±0.48	1.94±0.42	0.0001*
CAL (Clinical Attachment Loss) (mm)		2.75±0.46	0.8±0.01	0.0001*
BOP (Bleeding on Probing) (%)		43±19	15±7	0.0001*
Salivary HSP70 (ng/ml)		2.81±0.61	1.96±0.77	0.005*

* *p*< 0.05

**Table2:** Results of logistic regression analysis for determine the diagnostic power of salivary HSP70 in CP (Chronic Periodontitis)

Biomarker	Threshold	Odds ratio	95% Confidence interval	*p* Value
Salivary HSP70	1.96	10	1.2-3.4	0.045*

* *p*< 0.05

The results of spearman correlation analysis (Table 3) showed a positive correlation between salivary HSP70 and clinical periodontal index (PPD and CAL) suggesting that it could be used as a biomarker for diagnosis and monitoring therapeutic outcomes of periodontitis. In this analysis, no significant relationship was found between BOP and salivary HSP70 level.

**Table3:** Results of the spearman rank correlation test

Groups	Spearman correlation coefficient	*p* Value
PPD (Probing Pocket Depth ) & salivary HSP70	0.89	0.001*
CAL (Clinical Attachment Loss) & salivary HSP70	0.95	0.001*
BOP (Bleeding on Probing) & salivary HSP70	0.51	0.061

* *p*< 0.05

## Discussion

Currently, the diagnosis of periodontal disease depends mainly on clinical and radiographic factors. These parameters are valuable in identifying sign of past disease or confirming periodontal health, but do not provide sufficient evidence about patients who are at risk for future periodontal degeneration [ 19 ]. Salivary markers have been used in diagnosis of periodontal disease. Ideally, diagnostic tests must show high sensitivity and specificity [ 20 ]. Given the complex nature of CP, presenting a single sensitive and specific marker may not be accurate. Hence, a combination of two or more markers may provide a more precise evaluation of the periodontal disease. The interest in saliva as a diagnostic tool is growing. Both saliva and blood serum contain similar proteins, for this reason saliva is considered as a "body mirror" [ 15 ]. 

In the present study, HSP70 level in healthy individuals is significantly lower than moderate and severe periodontitis. Gokulanathan *et al*. [ 14 ] showed that increased salivary HSP60 levels in periodontitis could be a factor for systemic inflammation. Tsybikov *et al*. [ 13 ] showed that HSP70 levels in gingival fluid and serum samples were higher in subjects with periodontitis than in healthy subjects. The results of the study were consistent with those of the present study. In the study of Tabeta *et al*. [ 10 ], it was observed that, HSP60 level were significantly higher in patients with CP than in normal subjects.

HSPs area large family of proteins with highly conserved structure. They play a main role in the cell's core processes and act as a molecular adherent to other proteins [ 6 - 7 ]. HSPs have antioxidant and anti-inflammatory effects, help in early folding and refolding of proteins, protect the nucleus and lipid membrane against destruction, and prevent the cell from apoptosis. The HSP70 family is the most sensitive group of these proteins to temperature changes and has the most conserved structure [ 21 ].

The results of this study showed that the level of salivary HSP70 in patients with moderate and severe CP were significantly higher than that in healthy individuals. Moreover, the level of this biomarker was significantly correlated with periodontal clinical parameters, which indicated its potential for diagnosis and monitoring of response to treatment in patients with periodontitis. The reason of high salivary HSP70 level in patients with CP can be justified by identifying other important sources for salivary HSP70 including mucosal cells, gingival fluid, oral mucosal transduction and intraoral bleeding. Bacteria and other germs are also involved in the production of this protein. Salivary HSP70 causes bacterial uptake and accumulation and can bind to hydroxyapatite, which is the most important mineral on the tooth surface. Therefore, it seems that attaching the bacteria to the surface of the tooth can lead to plaque formation and periodontitis. HSP70 acts as a danger signal to release pro-inflammatory cytokines from several immune cells, and act as cytokine in the presence of immune cells [ 5 , 22 ].

In the current study, patients with mild periodontitis or gingivitis were not studied; therefore, it is not possible to investigate the relationship between the level of this biomarker and severity of the disease. Furthermore, increasing the salivary HSP70 level have been shown in other pathological conditions [ 23 - 24 ], which can reduce the specificity of this marker in the diagnosis of periodontal disease. Thus, further studies are necessary before salivary HSP70 level can be used as a reliable and effective diagnostic tool for screening and diagnosis of periodontal diseases.

## Conclusion

Based on the results of the current study, we emphasize the role of HSP70 in the pathogenesis of CP. Targeting HSP70 in periodontal treatments may improve outcomes. However, extensive studies should be conducted in future on the diagnostic value of this protein.
